# Nutritional management of metabolic disorders in neonates and infants in Saudi Arabia: consensus recommendations

**DOI:** 10.1186/s13023-025-03949-0

**Published:** 2025-11-17

**Authors:** Bedour Handoom, Eman Alohali, Hifa Elsagher, Lina Alohali, Naif Alhamed, Wafaa Alabyad

**Affiliations:** 1 King Faisal Specialist Hospital & Research Centre, Riyadh, Saudi Arabia; 2 Prince Sultan Military Hospital, Riyadh, Saudi Arabia; 3 National Guard Hospital, Riyadh, Saudi Arabia; 4 King Fahad Medical City, Riyadh, Saudi Arabia; 5 King Abdelaziz Hospital, Jeddah, Saudi Arabia

**Keywords:** Inherited errors of amino acid metabolism, Saudi arabia, Phenylketonuria, Homocystinuria, Propionic acidemia, Methylmalonic acidemia, Maple syrup urine disease, Glutaric acidemia type 1, Very-Long-Chain Acyl-CoA dehydrogenase

## Abstract

Inborn errors of metabolism (IEM) are inherited disorders affecting metabolism which can result in intellectual disability, cognitive impairment or death if left untreated. Nutritional management plays a major role in ensuring adequate growth, nutritional status and development, reducing levels of toxic metabolites, preventing deficiencies and avoiding catabolism in infants with IEM. The aim of this consensus is to provide recommendations for nutritional management of metabolic diseases in neonates and infants in Saudi Arabia, where a high frequency of IEM has been reported.

Consensus generation was performed using the Delphi method. Six metabolic dietitians from five hospitals across KSA formed the expert panel. Two face-to face meetings and one virtual meeting were conducted to generate consensus statements. Voting was conducted anonymously on SurveyMonkey to determine the level of agreement with each recommendation.

The expert panel reached consensus on 105 recommendations relating to the nutritional management of metabolic disorders, focusing on PA, MMA, GA1, PKU, MSUD, VLCAD, and HCU.

These recommendations will facilitate more consistent management of metabolic patients across Saudi Arabia and strive to highlight ongoing challenges faced by dietitians, patients, and caregivers. Future work should focus on outcomes associated with dietary management strategies in Saudi Arabia.

**Supplementary Information:**

The online version contains supplementary material available at 10.1186/s13023-025-03949-0.

## Background

Inborn errors of metabolism (IEM) are inherited disorders affecting metabolism which result from deficiencies in an enzyme, protein or transporter [1, 2]. Propionic acidaemia (PA) and methylmalonic acidaemia (MMA) are organic acidemias which result from defective mitochondrial enzymes propionyl CoA carboxylase and methylmalonyl CoA, respectively, leading to accumulation of toxic metabolic products which can cause acute metabolic decompensation [3]. Glutaric acidaemia type 1 (GA1) is a further organic acidemia resulting from a defective mitochondrial enzyme glutaryl-CoA dehydrogenase with a typical clinical presentation including macrocephaly and dystonia [4]. Phenylketonuria (PKU) is an aminoacidopathy disorder which results from deficiency of the phenylalanine hydroxylase enzyme and subsequent phenylalanine accumulation, causing brain damage [5]. Maple syrup urine disorder (MSUD) is a further aminoacidopathy disorder which is caused by dysfunction in the branched-chain α-ketoacid dehydrogenase (BCKAD) complex, leading to accumulation of the branched-chain amino acids (BCAA) leucine, isoleucine, and valine, causing damage to the brain and skeletal muscle [6]. Very-long chain acyl-CoA dehydrogenase deficiency (VLCADD) is a mitochondrial fatty acid oxidation disorder caused by deficiency in the very-long chain acyl-CoA dehydrogenase enzyme and leading to a range of clinical presentations from cardiomyopathy to hypoglycaemia, depending on age of onset [7]. Homocystinuria (HCU) is caused by deficiency in the cystathionine-β-synthase enzyme and typically results in thromboembolism and ophthalmic complications [8].

Globally it is estimated that inborn errors of metabolism occur at a prevalence of 50.9 per 100,000 live births, with amino acidurias being the most common at 14.7 per 100,000 live births [2]. The Eastern Mediterranean region, which includes Saudi Arabia, was reported to have the highest rate at 75.7 per 100,000 live births [2]. A meta-analysis revealed a pooled prevalence of PA ranging from 0.29 per 100,000 newborns in the Asia-Pacific region to 4.24 per 100,000 newborns in the Middle East and North Africa (MENA) region [9]. A further meta-analysis reported a pooled prevalence of MMA ranging between 0.79 per 100,000 newborns in the Asia-Pacific region to 6.04 per 100,000 newborns in the MENA region [10]. MSUD incidence has been reported to vary from 1 in 220,219 in the USA to 1 in 59,426 in Kuwait, according to an analysis of newborn screening program data [11]. PKU prevalence is estimated at 1 in 23,930 globally, ranging from 1 in 125,000 in Japan to 1 in 4,500 in Italy [12]. Newborn screening programs have reported prevalences of glutaryl-CoA dehydrogenase deficiency ranging from 1 in 100,800 newborns in Germany to 1 in 76,400 newborns in the USA, with an estimated global prevalence of 1 in 106,900 newborns [13]. Incidence of VLCADD has been reported to range from 1 in 32,000 live births in Taiwan to 1 in 400,000 live births in Saudi Arabia [14]. Finally, HCU prevalence is estimated at approximately 1 in 200,000 births globally [15].

High frequency of IEM has been reported in the Saudi Arabian population. A meta-analysis reported prevalences of PA from Saudi studies between 3.62 and 8.14 per 100,000 newborns [9]. Additionally, prevalence of MMA was reported between 6.45 and 14.48 per 100,000 newborns in a further meta-analysis [10]. In a review of patient medical records from Saudi ARAMCO, estimated incidence of IEM between 1983 and 2008 were 7 per 100,000 live births for classical PKU, 4 per 100,000 live births for PA, 2 per 100,000 live births for GA1, 7 per 100,000 live births for MSUD and 2 per 100,000 live births for HCU [16]. In a study in a single hospital in Jeddah, the reported incidence of IEM over a 5-year period were 1 in 8,248 live births for MMA, 1 in 16,497 live births for MSUD, 1 in 16,497 live births for HCU and 1 in 16,497 live births for VLCADD [17]. In a retrospective study of newborn screening in 139 hospitals across Saudi, an incidence of 1 in 14,090 was reported for PA, 1 in 14,245 for PKU, 1 in 15,500 for MMA, 1 in 15,816 for MSUD and 1 in 32,291 for GA1 [18]. In Riyadh, a retrospective study estimated an incidence of IEM of 8 in 100,000 for PA, 6 in 100,000 for MMA, 12 in 100,000 for HCU, 4 in 100,000 for PKU, 4 in 100,000 for MSUD and 2 in 100,000 for VLCAD over a 13-year period [19]. Finally, newborn screening in 3 Saudi hospital between 1995 and 1998, analysing 27,624 blood spots, reported prevalence of PKU of 1 in 9,208, MSUD of 1 in 13,812, GA1 of 1 in 13,812, MMA of 1 in 6,906 and PA of 1 in 27,624 newborns [20].

Nutritional management plays a major role in the management of metabolic disorders in infants. The goals of nutritional management include maintaining growth, nutritional status and development, reducing levels of toxic metabolites, correction of deficiencies and avoiding catabolism [21, 22]. Long-term health outcomes may be impacted if IEM are left untreated, including intellectual disability, cognitive impairment or death [23]. Nutritional guidelines from IEM have been developed by the Genetic Metabolic Dietitians International (GMDI) and the Southeast Regional Newborn Screening and Genetics Collaborative (SERC), the British Inherited Metabolic Diseases Group (BIMDG), and the European Network and Registry for Homocystinurias and Methylation Defects (E-HOD).

Challenges associated with IEM include the difficulties associated with dietary compliance, nutritional imbalances, metabolic decompensation, neurological implications, psychosocial impact, healthcare access and support as well as long-term complications [24, 25]. Parents of children with IEM often experience restrictions in many parts of their everyday lives, such as school, work, childcare, hobbies and spending time with friends; these restrictions are thought to be heightened with more severe disorders [25]. Dietary compliance affects children with IEM as well as their parents; in children avoiding restricted foods, being different from their peers and low self-esteem can be challenging; in parents the inability to breastfeed, feelings of guilt and anxiety surrounding their child’s diet are challenging [24].

The aim of this consensus is to provide recommendations for nutritional management of metabolic diseases in neonates and infants in Saudi Arabia, focusing on nutritional management below six months of age, above one year of age, weaning, emergency nutritional management, and sick day management.

## Methods

Consensus generation was performed using the Delphi method. Six metabolic dietitians from five hospitals across KSA formed the expert panel.

Two face-to-face meetings were conducted. The first meeting was held on the 9th of June 2023 in Utrecht, the Netherlands. The second meeting was held on the 29th of December 2023 in Cairo, Egypt. The aims of the meetings were to discuss the current protocols for nutritional management of metabolic diseases in neonates and infants, gather the perspectives of the expert panel on emergency and sick day management and determine appropriate nutrient requirements and dietary guidelines. The meeting and this consensus focused on the following seven metabolic diseases: PA, MMA, GA1, PKU, MSUD, VLCAD, and HCU.

A comprehensive literature review was undertaken to gather evidence pertaining to the nutritional management of these seven metabolic diseases. Search terms were: “Phenylketonuria”, “PKU”, “methylmalonic acidemia”, “MMA”, “propionic acidemia”, “PA”, “maple syrup urine disease”, “MSUD”, “glutaric aciduria type 1”, “GA1”, “very long-chain acyl-coenzyme A dehydrogenase deficiency”, “VLCAD”, “homocystinuria”, “HCU”, “inborn errors of metabolism”, “IEM”, “inherited metabolic disorders”, and “nutritional management”. Additionally existing recommendations from international guidelines, such as the GMDI, BIMDG, and E-HOD nutrition guidelines, were also reviewed for applicability to the nutritional management of KSA patients.

Following the face-to-face meetings and the detailed literature review, preliminary consensus recommendation statements were created. These preliminary statements were then reviewed by the expert panel and amended as necessary before voting was conducted using the SurveyMonkey platform. Statements were ranked as “strongly agree”, “agree”, “disagree”, “strongly disagree” or “neither agree or disagree” by the expert panel. For each statement, consensus was deemed achieved if 75% or more of the experts ranked the statement as “strongly agree” or “agree”.

## Results

### General principles of nutritional management

The expert panel reached consensus on all statements relating to the general principles of nutritional management of metabolic disorders (Table 1). Whilst early diagnosis and management is key, a collaborative approach and regular monitoring of patients is paramount.

**Table 1:** Consensus statements relating to general principles of nutritional management

	Statement	Consensus (%)
1.1	Early diagnosis and management are essential to prevent long-term sequalae or death as well as to ensure normal physical and mental development.	100
1.2	A collaborative approach involving healthcare professionals and caregivers is imperative.	100
1.3	Caregivers should be provided with a detailed treatment plan as well as emergency and sick day regimens.	100
1.4	Regular laboratory monitoring and evaluation of nutritional status are recommended.	100

### PA/MMA

The experts reached consensus on statements relating to the nutritional management of PA and MMA (Table 2).

**Table 2:** Consensus statements relating to nutritional management of PA/MMA

	Statement	Consensus (%)
Nutritional management in below 6 months of age		
2.1	At initial neonatal presentation, aggressive treatment and nutrition management is required to treat symptoms of metabolic acidosis, hyperammonaemia and dehydration.	100
2.2	Nutrition management involves the restriction of dietary propiogenic amino acids (isoleucine, valine, threonine, and methionine) and odd chain fatty acids.	100
2.3	Medical nutritional propiogenic amino acid-free-based formula can be used to achieve the recommended total protein intake.	83.3
2.4	Breastfeeding can be used to provide intact protein, with additional monitoring.	100
2.5	For stable individuals below 6 months of age recommended protein and energy requirements are as shown in Table 3.	100
2.6	Weekly follow-up is recommended in the first two months, then every two weeks until six months of age. At follow-up visits, a review of the following is recommended: • Protein and energy intake • Plasma amino acids and prealbumin levels • Vitamins, minerals, and micronutrient intake • Weight, length, and head circumference	100
Weaning practice		
2.7	Weaning can be initiated from 6 months of age if it is safe and possible to do so and taking into consideration weight and tolerance status of the patient.	100
2.8	Weaning with very-low protein vegetables and fruits is recommended.	100
2.9	For stable individuals aged 7 months to less than 12 months of age recommended protein and energy requirements are as shown in Table 3.	100
Nutritional management in above one year of age		
2.10	For stable individuals aged 1 to 8 years of age recommended protein and energy requirements are as shown in Table 3.	100
2.11	Follow-up is recommended every 3 months for individuals aged > 1 years. At follow-up visits, a review of the following is recommended: • Protein and energy intake • Plasma amino acids and prealbumin levels • Vitamins, minerals, and micronutrient intake • Weight, length/height, and head circumference, until 3 years of age	100
2.12	Introduce solid food as part of the total protein intake	100
Emergency nutrition management		
2.13	During acute illness, aggressive treatment and nutrition management is required to treat symptoms of metabolic acidosis, hyperammonaemia and dehydration.	100
2.14	During emergency nutritional management, protein intake should be restricted for no longer than 48 h or until ammonia levels are < 100µmol/l.	100
2.15	Use parenteral or enteral feeding, as appropriate, to supplement nutrients and fluids as well as administer medications.	83.3
2.16	Intact protein should be re-introduced by 50% for 24 h, then 100% after 24 h.	100
2.17	Ammonia levels should be monitored during emergency nutritional management to determine when it is safe to start feeding.	100
Sick day management		
2.18	Parents should be provided with a sick day management plan to initiate at home.	100
2.19	Natural protein intake should be restricted to 50% with mild illness, vaccination or minor operation for 24 h only.	100
2.20	If the patient has gastrointestinal symptoms of vomiting or diarrhea, intact protein should be stopped for 24 h and the patient should go directly to the emergency room.	100

**Table 3:** Protein and energy requirements by age [26]

Age	Intact Protein (g/kg/day)	Total Protein (g/kg/day)	Energy (kcal/kg/day)
0–6 months	0.91–1.52	1.52–1.82	Males: 72–109 Females: 72–108
7 months - <1 year	0.72–1.2	1.20–1.44	Males: 65–97 Females: 64–96
1–3 years	0.63–1.05	1.05–1.26	Males: 66–99 Females: 66–99
4–8 years	0.57–0.95	0.95–1.14	Males: 59–88 Females: 56–84

Used with permission of Elsevier Science & Technology Journals, from Nutrition management guideline for propionic acidemia: An evidence- and consensus-based approach, Jurecki E et al., 126, 2019; permission conveyed through Copyright Clearance Center, Inc.

### GA1

The experts reached consensus on statements relating to the nutritional management of GA1 (Table 4).

**Table 4:** Consensus statements relating to nutritional management of GA1

	Statement	Consensus (%)
Nutritional management in below 6 months of age		
3.1	Nutritional management involves a reduced lysine and tryptophan diet.	83.3
3.2	Medical nutritional lysine-free low tryptophan amino acid-based formula can be used to achieve the recommended total protein intake.	83.3
3.3	Supplementation with L-carnitine and riboflavin is recommended.	100
3.4	For stable individuals below 6 months of age recommended protein and energy requirements are as shown in Table 5.	100
3.5	Weekly follow-ups are recommended in the first two months, then every two weeks until six months of age. At follow-up visits, a review of the following is recommended: • Protein and energy intake • Plasma amino acids and prealbumin levels • Vitamins, minerals, and micronutrient intake • Weight, length/height, and head circumference	100
Weaning practice		
3.6	Weaning can be initiated from 6 months of age if it is safe and possible to do so and taking into consideration weight and tolerance status of the patient.	100
3.7	Weaning with very-low protein vegetables and fruits is recommended.	100
3.8	Where lysine and tryptophan content of food may be difficult to calculate, total protein intake is calculated instead.	100
3.9	For stable individuals aged 7 months to less than 12 months of age recommended protein and energy requirements are as shown in Table 5.	100
Nutritional management in above one year of age		
3.10	For stable individuals aged 1 to 6 years recommended protein and energy requirements are as shown in Table 5.	100
3.11	Three to six monthly follow-ups are recommended for 1–6 years old. At follow-up visits, a review of the following is recommended: • Protein and energy intake • Plasma amino acids and prealbumin levels • Vitamins, minerals, and micronutrient intake • Weight, length/height, and head circumference	83.3
3.12	Introduce solid food as part of the total protein intake	100
3.13	After 6 years of age, diet can be liberalised, and medical nutritional formulas may be discontinued. Supplementation with L-carnitine should continue.	100
Emergency nutrition management		
3.14	If symptoms of acute crisis are present (diarrhoea, vomiting, fever, and neurologic symptoms), emergency treatment should be initiated immediately and aggressively.	100
3.15	During emergency nutritional management, natural protein intake should be restricted by 50% for up to 24 h, then re-introduced gradually to 100%.	100
3.16	Protein-free modulars should be administered to provide additional calories from carbohydrates or fats.	100
Sick day management		
3.17	Natural protein intake should be restricted to 50% with mild illness, vaccination or minor operation for 24 h only.	100

**Table 5:** Protein and energy requirements by age [27]

	Lysine (natural protein), mg/kg/day	Amino acid mixtures (protein), g/kg/day	Energy, kcal/kg/day
0–6 months	100	1.3–0.8	100 − 80
7–12 months	90	1.0-0.8	80
1–3 years	80 − 60	0.8	94 − 81
4–6 years	60 − 50	0.8	86 − 63

Used with permission of John Wiley & Sons - Books, from Recommendations for diagnosing and managing individuals with glutaric aciduria type 1: Third revision, Boy N et al., 46, 2022; permission conveyed through Copyright Clearance Center, Inc.

### PKU

The experts reached consensus on statements relating to the nutritional management of PKU (Table 6).

**Table 6:** Consensus statements relating to nutritional management of PKU

	Statement	Consensus (%)
Nutritional management in below 6 months of age		
4.1	Nutritional management involves a phenylalanine-restricted diet and tyrosine supplementation if needed.	83.3
4.2	Medical nutritional phenylalanine-free formula can be used to achieve the recommended total protein intake.	83.3
4.3	Breastfeeding can be used to provide phenylalanine and intact protein.	83.3
4.4	For stable individuals below 6 months of age recommended protein, PHE and TYR requirements are as shown in Table 7.	100
4.5	Weekly follow-ups are recommended in the first two months, then every two weeks until six months of age. At follow-up visits, a review of the following is recommended: • Protein and energy intake • Plasma amino acids and prealbumin levels • Vitamins, minerals, and micronutrient intake • Weight, length/height, and head circumference	100
4.6	Introduce solid food as part of the total protein intake	100
Weaning practice		
4.7	Weaning can be initiated from 6 months of age.	100
4.8	Nutritional management involves calculating the amount of phenylalanine intake based on protein requirement.	100
4.9	For stable individuals aged 6 months to less than 12 months of age recommended protein, PHE and TYR requirements are as shown in Table 7.	100
Nutritional management in above one year of age		
4.10	For stable individuals aged 1–4 years recommended protein, PHE and TYR requirements are as shown in Table 7.	100
4.11	Stable individuals aged 1 to 8 years should be followed-up every 3 months.	100
High PHE Level Management		
4.12	If PHE levels are high, then 0 mg PHE is given for 24–48 h.	83.3

**Table 7:** Protein, PHE and TYR requirements by age [28]

	Protein, g/kg/day	PHE, mg/day	TYR, mg/day
0 to < 3 months	2.5-3.0	130–430	1100–1300
3 to < 6 months	2.0–3.0	135–400	1400–2100
6 to < 9 months	2.0-2.5	145–370	2500–3000
9 to < 12 months	2.0-2.5	135–330	2500–3500
1 to < 4 years	1.5–2.1	200–320	2800–3500

Used with permission of Elsevier Science & Technology Journals, from Updated, web-based nutrition management guideline for PKU: An evidence and consensus based approach, Singh RH et al., 118, 2016; permission conveyed through Copyright Clearance Center, Inc. PHE, phenylalanine; TYR, tyrosine

### MSUD

The experts reached consensus on statements relating to the nutritional management of MSUD (Table 8).

**Table 8:** Consensus statements relating to nutritional management of MSUD

	Statement	Consensus (%)
Nutritional management in below 6 months of age		
5.1	At initial presentation, BCAA-free protein formula as well as isoleucine and valine supplementation should be provided.	100
5.2	Frequent feedings should be given to ensure adequate energy levels.	100
5.3	BCAA protein should be reintroduced gradually.	100
5.4	Treatment should be individualised based on the severity of the case, plasma amino acid levels and specific patient needs.	100
5.5	Breastfeeding can be used to provide BCAA, with careful monitoring.	100
5.6	For individuals below 6 months of age recommended levels of BCAA, protein, energy, and fluids are as shown in Table 9.	100
5.7	Individuals below 6 months of age should be followed-up weekly. The amino acid profile should be checked, where available.	100
Weaning practice		
5.8	Weaning can be initiated from 6 months of age.	100
5.9	Weaning with fruits and vegetables or low protein product (rice or cereal) is recommended.	100
5.10	For individuals aged 7 months to 12 months of age recommended levels of BCAA, protein, energy, and fluids are as shown in Table 9.	100
Nutritional management in above one year of age		
5.11	For individuals above the age of 1 year, it is recommended to eliminate all high leucine content foods. The diet should consist of fruits and vegetables or low protein product.	83.3
5.12	Each 1 g of protein is equal to 60 mg of leucine.	100
5.13	For individuals aged 1 to 3 years recommended levels of BCAA, protein, energy, and fluids are as shown in Table 9.	100
5.14	Individuals above the age of 1 year should be followed-up every 3 to 6 months. At follow-up, levels of B12, zinc, selenium, pre-albumin, iron, plasma amino acids, and complete blood count should be assessed.	100
Emergency and sick day management		
5.15	Caregivers should be aware of the signs and symptoms of imbalance in BCAA and elevated leucine.	100
5.16	Emergency or sick day management should be initiated at home and continued once admitted to hospital.	100
5.17	The emergency or sick day regimen should consist of 0% BCAA, whilst supplementing valine and isoleucine, followed by 50% BCAA then 100% BCAA, based on the amino acid profile.	100
5.18	Following emergency or sick day management, amino acid levels should be re-assessed to ensure BCAA levels are normalised.	100

**Table 9:** Protein, BCAA, energy, and fluid requirements by age [29]

	LEU, mg/kg	ILE, mg/kg	VAL, mg/kg	Protein, g/kg	Energy, kcal/kg	Fluid, mL/kg
0–6 months	40–100	30–90	40–95	2.5–3.5	95–145	125–160
7–12 months	40–75	30–70	30–80	2.5-3.0	80–135	125–145
1–3 years	40–70	20–70	30–70	1.5–2.5	80–130	115–135
4–8 years	35–65	20–30	30–50	1.3-2.0	50–120	90–115

Used with permission of Elsevier Science & Technology Journals, from Nutrition management guideline for maple syrup urine disease: an evidence- and consensus-based approach, Frazier DM et al., 112, 2014; permission conveyed through Copyright Clearance Center, Inc. ILE, isoleucine; LEU, leucine; VAL, valine

### VLCAD

The experts reached consensus on statements relating to the nutritional management of VLCAD (Table 10).

**Table 10:** Consensus statements relating to nutritional management of VLCAD

	Statement	Consensus (%)
Nutritional management in below 6 months of age		
6.1	Initiate treatment if newborn screening is positive for VLCAD or whenever there is a confirmed diagnosis.	83.3
6.2	Treatment should involve cessation of breastfeeding, initiation of medium chain triglyceride (MCT) formula, omega 3 supplementation and avoiding long intervals between feeds.	100
6.3	For individuals aged 0–6 months recommended fat and energy requirements are as shown in Table 11.	100
6.4	Individuals below 6 months of age should be followed-up monthly. Follow-up should include a nutritional assessment and comprehensive laboratory tests, including vitamins and minerals.	83.3
Weaning practice		
6.5	Weaning can be initiated from 6 months of age.	100
6.6	Weaning should involve limited intake of dietary fats and continuation of MCT formula and MCT oil.	83.3
6.7	For individuals aged 7–12 months recommended fat and energy requirements are as shown in Table 11.	100
6.8	Individuals aged 6–12 months should be followed-up every 3 months. Follow-up should include a nutritional assessment and comprehensive laboratory tests, including vitamins and minerals.	83.3
Nutritional management in above one year of age		
6.9	For individuals aged 1–3 years recommended fat and energy requirements are as shown in Table 11.	100
6.10	For individuals aged 1 year and older, it is recommended to continue a low LCT diet and MCT supplementation.	100
6.11	For individuals aged 1 year and older, C7 can be used, if available and safe to be used.	100
6.12	Individuals aged 1 year and older should be followed-up every 3–6 months. Follow-up should include a nutritional assessment and comprehensive laboratory tests, including vitamins and minerals.	83.3
Emergency and sick day management		
6.13	For mild illness, sick day management at home should consist of frequent high carbohydrate meals, reduced fasting intervals and MCT supplementation.	100
6.14	If individuals are admitted to hospital, 10% IV dextrose with electrolytes at 1.5x maintenance fluids is recommended if they are not able to consume adequate energy.	100

**Table 11:** Fat and energy requirements by age [30]

Age	Severity	Total fat, % of total energy	Long-chain fat, % of total energy	Medium-chain fat, % of total energy
0–6	Severe	40–55	10–15	30–45
	Moderate		15–30	10–30
	Mild		30–55	0–20
7–12	Severe	35–45	10–15	25–30
	Moderate		15–30	10–25
	Mild		30–40	0–10
1–3	Severe	30–40	10–15	10–30
	Moderate		20–30	10–20
	Mild		20–40	0–10

Used with permission of Elsevier Science & Technology Journals, from Nutrition management guideline for very-long chain acyl-CoA dehydrogenase deficiency (VLCAD): An evidence- and consensus-based approach, Van Calcar SC et al., 131, 2020; permission conveyed through Copyright Clearance Center, Inc.

### HCU

The experts reached consensus on statements relating to the nutritional management of HCU (Table 12).

**Table 12:** Consensus statements relating to nutritional management of HCU

	Statement	Consensus (%)
Nutritional management in below 6 months of age		
7.1	Initiate treatment if newborn screening is positive for HCU or whenever there is a confirmed diagnosis.	100
7.2	For individuals below 6 months of age, treatment should involve supplementation with methionine-free formula.	100
7.3	Individuals below 6 months of age should be followed-up weekly. Follow-up should involve the monitoring of plasma methionine and homocysteine levels. Formula volume should be adjusted to maintain target plasma levels of methionine and homocysteine.	100
7.4	Plasma target levels of homocysteine are: 60–100 µmol/L total homocysteine and ≤ 10 µmol/L.	
Weaning practice		
7.5	Weaning can be initiated from 6 months of age.	100
7.6	Weaning should involve low protein foods, low protein prescribable products and methionine-free formula.	100
7.7	Individuals aged 6–12 months should be followed-up every 3–6 months. Feeding should be reviewed at follow-up.	100
Nutritional management in above one year of age		
7.8	Introduce solid food as part of the total protein intake.	100
7.9	Individuals aged 1 year and older should be followed-up every 3–6 months. Feeding should be reviewed at follow-up.	100

### Role of additions to infant formula for nutritional benefit

Consensus was reached on all statements relating to the role of additions to infant formula for nutritional benefit (Table 13). Multivitamin and mineral supplementation as well as essential fatty acid supplementation are recommended.

**Table 13:** Consensus statements relating to the role of additions to infant formula for nutritional benefit

	Statement	Consensus (%)
8.1	Metabolic patients with restricted diets should have multivitamin and mineral supplementation to prevent macronutrient and micronutrient deficiencies.	100
8.2	Essential fatty acid supplementation can be used for all metabolic patients.	100
8.3	Galactooligosaccharides (GOS), fructooligosaccharides (FOS), Docosahexaenoic acid (DHA) and arachidonic acid (ARA) are not generally used unless the patient is severely unwell.	83.3

### Challenges in dietary management of metabolic disorders

Consensus was reached on all statements relating to the challenges faced in the dietary management of metabolic disorders (Table 14). Adherence to diet and meeting nutritional adequacy are key challenges faced by metabolic patients whilst the lack of unified guidelines and lack of authority are challenges faced by clinical dietitians in KSA.

**Table 14:** Consensus statements relating to challenges in dietary management of metabolic disorders

	Statement	Consensus (%)
9.1	Poor adherence to diet can result in complications and frequent hospitalisations.	100
9.2	Attaining nutritional adequacy and meeting growth requirements are key challenges in the management of metabolic disorders.	100
9.3	Individuals with metabolic disorders and their caregivers experience significant psychological burden and social isolation.	83.3
9.4	An unmet need in the dietary management of metabolic disorders is a lack of unified guidelines for clinical dietitians in KSA.	100
9.5	A lack of authority of clinical dietitians to order appropriate testing relevant to metabolic diseases is a significant challenge.	100

### Caregiver counselling

Consensus was reached on all statements relating to caregiver counselling (Table 15). Adequate counselling using a counselling checklist following patient diagnosis as well as continuous communication are essential for the successful nutritional management of these patients.

**Table 15:** Consensus statements relating to caregiver counselling

	Statement	Consensus (%)
10.1	Following a diagnosis, caregivers should be counselled using a counselling checklist.	100
10.2	The counselling checklist should include the nature of the disease, manifestations of the disease, symptoms of metabolic decompensation, the importance of diet and adherence to dietary restrictions, preparation of medical nutritional formula as well as emergency and sick day management regimens.	100
10.3	Continuous communication between caregivers and the metabolic centre is imperative, including a call or messaging helpline.	100

## Discussion

### General principles of nutritional management

Early diagnosis and management of IEM are essential. Newborn screening programs for IEM enable early detection, before the development of symptoms, and can improve quality of life and life expectancy is individuals with IEM as well as prevent death, disability or intellectual disability [31].

A collaborative approach involving healthcare professionals and caregivers is imperative to successful nutritional management of IEM. Multidisciplinary care of IEM patients should involve a clinician and a dietitian in a cooperative partnership with nurses, physiotherapists, social workers, and psychologists [32, 33]. Additionally, care should be centred around the patient with active and on-going communication between the patient, their family, and the multidisciplinary healthcare team [32].

Caregivers should be provided with a detailed treatment plan as well as emergency and sick day regimens. The treatment plan should include details of specific medical formulas, how to prepare and store them, breastfeeding recommendations, supplements, foods which can be consumed, foods to be avoided as well as monitoring [34]. Since children with IEM are a greater risk of hypoglycaemia or metabolic decompensation during sick days, parents should be instructed on the necessary steps to take and written instructions regarding an emergency regimen including recommended fluid, glucose polymers recipe, contact numbers and recipes [35].

Regular laboratory monitoring and evaluation of nutritional status are recommended in all patients with IEM. According to the GMDI guidelines frequent monitoring of biochemicals, growth as well as clinical symptoms are essential to ensure levels of toxic metabolites remain low, patient nutritional status and growth are adequate and the patient is showing no signs of metabolic decompensation or other complication [36–39]. Biochemicals which require monitoring for each disease are detailed in the Appendix 1 and references ranges in children are shown in Appendix 2 of the supplementary file.

### PA/MMA

#### Nutritional management in below 6 months of age

The GMDI recommends aggressive treatment and nutrition management at initial neonatal presentation [37]. Acute management includes intravenous administration of high calorie and high carbohydrate fluids (additional intake of 100–120 kcal/kg/day; 10% dextrose) and lipids to prevent catabolism as well as insulin to correct hypoglycaemia and hyperglycaemia [40]. Nutrition management in infants below 6 months of age also involves the restriction of dietary propiogenic amino acids (isoleucine, valine, threonine, and methionine) and odd chain fatty acids by reducing the intake of intact protein [37]. Intact protein at 60–100% of the normal daily recommended amount is recommended [41], since over-restriction of protein is thought to affect patient growth [42]. For patients who are not able to achieve their daily recommended intake of intact protein, medical nutritional propiogenic amino acid-free-based formula can be used [41].

In infants below 6 months, breastfeeding can be used to provide intact protein, however, there is the need for additional monitoring. The GMDI reached consensus on use of breast milk in PA patients [37]. Experience of breastfeeding in infants with MMA has also been described, with breastfeeding being well tolerated in individuals with MMA for up to 18 months [43]. For stable individuals below 6 months of age recommended protein and energy requirements are as shown in Table 3, according to the GMDI [26]. These values represent 60–100% intact protein, 100–120% total protein and 80–120% estimated energy requirements from the Institute of Medicine’s Dietary Reference Intakes [44] and aim to achieve adequate protein and energy intake to ensure growth and anabolism whilst avoiding high intake of propiogenic amino acids and accumulation of toxic compounds [26].

Regular patient follow-up is recommended for infants with PA and MMA, including the monitoring of biochemicals and nutritional status [37]. The GMDI recommends weekly to monthly follow-up in children under one year of age to assess diet and nutrient intake, weight, length, head circumference, plasma amino acids, and pre-albumin [26]. The trans-European guidelines recommend follow-up is determined on an individual basis, taking into account patient factors and treatments [45].

### Weaning practice

Weaning, with very-low protein vegetables and fruits, is recommended from 6 months of age if it is safe and possible to do so and taking into consideration weight and tolerance status of the patient [45]. Very-low protein vegetables and fruits include apples, grapes, peaches, pears, lettuce, mushroom, pepper, and asparagus [46]. Tube feeding maybe recommended in severe cases as well as speech and language therapy which can help to improve potential feeding problems [45]. For stable individuals aged 7 months to less than 12 months of age, recommended protein and energy requirements are as shown in Table 3, as per the GMDI [26].

### Nutritional management in above one year of age

For stable individuals aged 1 to 8 years recommended protein and energy requirements are shown in Table 3, as per the GMDI [26]. These values represent 60–100% intact protein, 100–120% total protein and 80–120% estimated energy requirements from the Institute of Medicine’s Dietary Reference Intakes [44] and aim to achieve adequate protein and energy intake to ensure growth and anabolism whilst avoiding high intake of propiogenic amino acids and accumulation of toxic compounds [26]. The Saudi experts recommend follow-up every 3 to 4 months for individuals aged > 1 to 7 years to assess diet and nutrient intake, weight, length, head circumference (for patients up to 3 years), plasma amino acids, and pre-albumin [26].

### Emergency nutrition management

During acute illness, aggressive treatment and nutrition management is required to treat symptoms of metabolic acidosis, hyperammonaemia, and dehydration. Similar to neonatal presentation, intravenous administration of high calorie (additional intake of 100–120 kcal/kg/day), high carbohydrate fluids (10% dextrose) and lipids to prevent catabolism as well as insulin to correct hypoglycaemia and hyperglycaemia [40]. The use of high calories provides additional energy to promote anabolism [45]. Fluid infusion at 150 ml/kg/24 hours is recommended to treat dehydration and intravenous bicarbonate is given to treat metabolic acidosis [45].

During emergency nutritional management, protein intake should be restricted for no longer than 48 h. Since amino acids are essential building blocks for the body to grow and develop, protein intake should only be stopped temporarily, for a maximum of 24 to 48 h [47]. In cases where protein intake is restricted for too long or inadequate, the patient is at greater risk of nutrient deficiency, impaired growth, impaired immunity as well as bone fractures [48]. Enteral feedings should be promptly started following this period of protein restriction and use of parenteral feeding is recommended with severe illness or where enteral feeding is not possible [45]. Intact protein should be re-introduced gradually to 50% natural protein then 100% natural protein each day, as tolerated by the patient [37].

Ammonia levels should be monitored during emergency nutritional management to determine when it is safe to start feeding. High ammonia levels can lead to nervous system damage and even death if left untreated.

### Sick day management

Parents should be provided with a sick day management plan to initiate at home [26]. Such a plan might include reduced propiogenic amino acids and additional medical foods in order to provide sufficient calories and avoid complications necessitating hospitalisation [49]. Natural protein intake should be restricted to approximately 50% with mild illness, vaccination or minor operation [37]. In such situations, a resulting fever, dehydration or reduced energy intake may put the patient at risk of metabolic decompensation [45]. Since immune deficits are thought to occur in 30–65% of PA and MMA cases, responses to vaccination may be unpredictable and therefore patients require careful monitoring or cautious administration of vaccines [50]. If a patient develops gastrointestinal symptoms, intact protein should be stopped for 24 h, and the patient should go directly to the emergency room for emergency nutrition management [37].

### GA1

#### Nutritional management in below 6 months of age

Nutritional management of GA1 involves a reduced lysine and tryptophan diet. A low lysine diet reduces the accumulation of toxic metabolites whilst ensuring adequate micronutrient intake [27]. In a German study of 33 infants identified via NBS and followed prospectively until 6 years of age, a low lysine diet led to normal weight gain but reduced body length in asymptomatic individuals with GA1 as well as normal biochemical parameters [51].

Medical nutritional lysine-free low tryptophan amino acid-based formula can be used to achieve the recommended total protein intake and supplementation with L-carnitine are recommended by the GA1 guideline development group [27]. Supplementation with lysine-free tryptophan-reduced amino acids in conjunction with a low-lysine diet and carnitine supplementation has shown beneficial effects on the development and uptake of essential micronutrients in children with asymptomatic GA1 [51]. A meta-analysis of outcomes of patients identified by NBS and undergoing maintenance treatment of low-lysine diet, lysine-free, trytophan-reduced, arginine-fortified amino acids and L-carnitine supplementation showed positive effects on neurological outcomes, including mortality, motor development and complex movement disorder [52]. A further analysis of a cohort of patients identified by NBS and treated with lysine-free, arginine-enriched formula in addition to L-cartinine showed normal growth, cognitive function and motor development, preventing 90% of striatal injuries in the first two years of life [53].

For stable individuals below 6 months of age recommended protein and energy requirements are shown in Table 5, as recommended by the GA1 guideline development group [27]. Clinical monitoring is recommended every 3 months for those aged less than 1 year and includes weight, length or height and head circumference, lysine, protein, fat and calorie intake, clinical status and laboratory parameters [27].

### Weaning practice

Weaning can begin at 6 months if considered safe and appropriate and taking into consideration weight and tolerance status of the patient. During weaning, patients face the challenge of different protein sources and variation in lysine content of different foods [54]. Therefore, proper dietary management is essential during weaning to prevent complications and support healthy development. Weaning with very-low protein vegetables and fruits is recommended [55].

Where lysine content of different foods may be difficult to calculate, total protein intake may be calculated instead. According to the Metabolic Dietitians National Centre for Inherited Metabolic Disorders, dietary management should involve a low protein diet [56]. However, in general, dietary treatment of GA1 has moved from a protein restricted diet to a low lysine and tryptophan diet since calculating lysine intake is much more accurate [57, 58]. The GA1 guideline development group only recommend a low-protein diet from the age of 6 years [27].

For stable individuals aged 7 months to less than 12 months of age recommended protein and energy requirements are shown in Table 5, as recommended by the GA1 guideline development group [27]. In a cohort of infants with GA1 from the USA, who were identified using NBS and for whom dietary management involved a restricted natural protein intake of 1.0–1.3 g/kg per day, supplemented by a lysine-free, arginine-enriched metabolic formula, reduced striatal degeneration while supporting normal growth and development was observed compared to GA1 patients identified by NBS and treated with natural protein restriction or those with neither NBS nor dietary management [53].

### Nutritional management in above one year of age

For stable individuals aged 1 to 6 years recommended protein and energy requirements are shown in Table 5, as recommended by the GA1 guideline development group [27]. According to recent guidelines, cohort studies and dietitian surveys, protein intake should be accurately controlled, typically between 1.0 and 1.3 g/kg/day, to minimize lysine and tryptophan intake while ensuring adequate nutrition [27, 53, 59].

Routine six-monthly follow-ups are critical for assessing the health and nutritional status of children aged 1–6 years with GA1. Key areas for review during these follow-up visits include protein and energy intake, vitamins, minerals, and micronutrient intake, anthropometric measurements, plasma amino acids, and pre-albumin. Confirming adherence to dietary restrictions and ensuring sufficient energy intake to prevent catabolism are vital [53, 60, 61]. Evaluating essential nutrient intake is also important to prevent deficiencies, which are common with dietary limitations [27, 57, 62]. Tracking weight, height or length, and head circumference to monitor growth, is essential for ensuring the child is developing appropriately for their age [51, 53, 61].

After the age of 6 years, dietary restrictions can be gradually relaxed, lysine and tryptophan limitations may be eased, and medical nutritional formulas can be phased out [27, 53, 59]. However, continued L-carnitine supplementation remains essential to support metabolic function and prevent the buildup of toxic metabolites. The long-term effects of dietary liberalization and when to relax dietary management in GA1 patients are still under investigation [57], but existing evidence supports that it can be done safely with medical oversight [27, 53, 63].

### Emergency nutrition management

During acute crises, immediate and intensive treatment is essential to prevent irreversible damage caused by the buildup of toxic metabolites. In a meta-analysis, delays in the initiation of emergency treatment have been shown to increase the risk of acute onset movement disorder in individuals with GA1 who were identified by newborn screening [52]. During acute metabolic crisis, restricting natural protein intake by 50% for up to 24 h, followed by gradual reintroduction over 48 to 72 h, is recommended by the GA1 guideline development group [63]. Additional caloric intake from carbohydrate modulars is also important to prevent catabolism and meet energy demands [58–60].

### Sick day management

Since fever, illness or surgery are known to precipitate acute crises, appropriate management during these times is essential [27]. Experts recommend a formula containing no natural protein for the first 24 h of admission to hospital. Strauss et al. recommend restricting natural protein to 0.5–0.6 g/kg/day, a 50% reduction, as well as use of medications to control fever, antibiotics to control infection and greater frequency of feeding [53].

### PKU

#### Nutritional management in below 6 months of age

Following a diagnosis, a low-phenylalanine diet is critical for PKU management and should begin promptly to prevent cognitive and developmental complications [28, 64]. Phenylalanine-free or low-phenylalanine medical formulas are prescribed to meet the protein needs of infants with PKU, supporting healthy growth while maintaining safe phenylalanine levels [28, 65]. Breast milk is low in phenylalanine [66], therefore, breastfeeding can be continued safely in infants with PKU, accompanied by a low- phenylalanine formula to ensure adequate phenylalanine and intact protein intake. This approach, endorsed by European guidelines, has demonstrated safety and potential benefits for growth and neurodevelopment [67].

For stable individuals below 6 months of age recommended protein, PHE and TYR requirements are shown in Table 7, as recommended by the GMDI. The goal of nutritional management in individuals with PKU is to maintain a plasma phenylalanine concentration between 120 and 360 µmol/L [68]. The Saudi experts recommend regular follow-up, with weekly visits during the first two months and bi-weekly visits thereafter until six months. GMDI guidelines recommend assessment at the clinic weekly to every 3 months including weight, length or height, and head circumference. Additionally, PHE and TYR should be routinely monitored every week to every two weeks [39]. At follow-up visits, a review of protein and energy intake, vitamins, minerals, and micronutrient intake and growth measurements is recommended. Regular review of protein and energy intake is important to ensure the infant’s nutritional needs are being met without exceeding safe phenylalanine levels [28, 65]. Monitoring of vitamins, minerals, and micronutrient intake is essential for the prevent deficiencies and support overall health [65]. Tracking weight, length/height, and head circumference are recommended to monitor the patients’ growth and development [28].

### Weaning practice

Weaning for infants can be initiated from 6 months, according to the GMDI [28], although some centres are reported to wean as early as 17 weeks [66]. Weaning typically involves fruit and vegetables containing 75 mg phenylalanine per 100 g of food or less as first foods, such as apples, carrots, pears, and sweet potato [66]. Nutritional management involves calculating phenylalanine intake relative to protein intake and monitoring fruit and vegetable consumption [28, 69, 70]. Guidelines and recommendations state that fruits and vegetables containing 75 mg phenylalanine per 100 g of food or less can be consumed without restriction [71, 72]. However, recent evidence suggests that fruits and vegetables containing 76–100 mg of phenylalanine per 100 g may be included in the PKU diet, depending on disease severity, but their effect on blood phenylalanine levels should be carefully monitored [69, 70].

For stable infants aged 6–12 months, protein needs should be met through a combination of natural protein and protein substitutes to support proper growth and development [28, 65]. Recommended protein, PHE and TYR requirements are shown in Table 7, as per the GMDI. Phenylalanine intake should be meticulously calculated and monitored to maintain target blood phenylalanine levels. Tyrosine supplementation may also be required if concentration in the blood is low, as individuals with PKU lack the ability to convert phenylalanine to tyrosine [28].

### Nutritional management in above one year of age

For stable individuals aged 1–4 years recommended protein, PHE and TYR requirements recommended are shown in Table 7, as per the GMDI. Children with PKU often require a higher protein intake than current standard recommendations, with research suggesting an average protein requirement of around 1.85 g/kg/day for children with PKU, exceeding the previously recommended range of 1.14–1.33 g/kg/day [73]. Since protein intake for PKU management primarily comes from protein substitutes, this should be carefully prescribed to meet each child’s metabolic needs [74]. The target blood phenylalanine concentration for children aged 0–12 years is 120–360 µmol/L, as recommended by European guidelines [75]. Additionally, phenylalanine tolerance tends to increase with age, especially during puberty, reaching its peak toward the end of adolescence [69]. Tyrosine levels should be regularly monitored to stay within the target range. A retrospective cohort study of PKU patients from Portugal found that the introduction of glycomacropeptide (GMP) as a protein source can elevate blood tyrosine levels, however, further research on this approach is needed [76].

European guidelines emphasize that regular nutritional, clinical, and biochemical follow-up is necessary for all PKU patients, irrespective of the treatment approach. The frequency of follow-up should be individualized based on age, treatment adherence, and clinical condition [75]. Additionally, regular reassessment of natural protein tolerance is important to avoid unnecessary dietary restriction as well as to ensure optimal intake [77].

### Sick day management

For sick day management, a decrease in protein intake is recommended by the Saudi experts. Although there is little research into the management of PKU patients during sick days, it is thought that a reduced appetite during illness generally results in lower protein intake and there is no requirement to formally remove natural protein from the patient’s diet [66].

### MSUD

#### Nutritional management in below 6 months of age

For infants with MSUD, the GMDI recommends a BCAA-free protein formula supplemented with isoleucine and valine to prevent deficiencies and manage plasma leucine levels [29, 78, 79]. Additionally, frequent feedings are advised to maintain energy levels and prevent catabolism, which can worsen the condition [78, 79]. Following a diagnosis, natural protein should be reintroduced slowly to monitor tolerance and prevent metabolic imbalances [80, 81]. Treatment in infants should be personalized according to the case’s severity and patient-specific needs, involving regular plasma BCAA level monitoring and dietary adjustments as needed [29, 81, 82].

Breastfeeding, which is encouraged by guideline recommendations, provides intact protein and BCAAs, but requires careful monitoring to ensure that BCAA levels remain within safe limits [29, 81]. For individuals below 6 months of age recommended levels of BCAA, protein, energy, and fluids are shown in Table 9, as recommended by the GMDI [29]. MSUD management aims is to maintain plasma leucine concentrations between 100 and 200 µmol/L for infants and children under 5 years. Adequate energy intake, approximately 125 kcal/kg/day, and sufficient protein intake are crucial to support growth and prevent catabolism [78, 81, 83]. Bimonthly follow-ups are recommended for infants under 6 months of age, with routine checks of the amino acid profile to ensure effective management and dietary adjustments [29, 36, 81, 82].

### Weaning practice

Saudi experts recommend weaning from 6 months of age with fruits and vegetables or low protein rice or cereal. The National Centre for Inherited Metabolic Disorders in Ireland suggests introducing protein-free foods as first foods between 4 and 6 months of age [34]. For individuals aged 7 months to 12 months of age recommended intake levels of BCAA, protein, energy, and fluids are shown in Table 9, as per the GMDI [29]. The aim is to maintain leucine plasma levels of 75–300 µmol/L and isoleucine and valine of 200–400 µmol/L [29]. A retrospective cohort study demonstrated that leucine levels less than 200 µmol/L are associated with better cognitive outcomes compared to higher leucine levels [84].

### Nutritional management in above one year of age

For individuals above the age of 1 year, foods high in leucine should be restricted, and medical foods free of BCAAs should be incorporated. The diet should mainly include fruits, vegetables, and low-protein foods to help regulate leucine levels [29, 78, 85]. Leucine supplementation is often not required since adequate levels of found in breast milk and formula [86]. Supplemental valine and isoleucine, however, are frequently required to balance the limited leucine intake and maintain suitable plasma levels of these amino acids [78, 79, 87]. A 10% oral solution can be made by dissolving 1 g of valine and isoleucine in 100 ml of water. If supplementation is not available, medical food designed for the treatment of isovaleric acidaemia that are devoid of leucine but contains valine and isoleucine, can be used.

Protein intake should be individualized based on the patient’s tolerance, with a general guideline of about 1 g of protein daily, equivalent to 60 mg of leucine [29, 78, 87]. The Saudi experts recommend that only dietary leucine should be counted since valine and isoleucine content of the food is approximately half that of leucine and therefore the patient will not consume too much valine and leucine if they match the prescribed leucine intake.

For individuals aged 1 to 3 years recommended levels of BCAA, protein, energy, and fluids are shown in Table 9, as per GMDI [29]. For children aged 1 to 3 years, experts recommend close monitoring and adjustment of BCAAs, protein, energy, and fluid intake to support healthy growth and avoid metabolic decompensation [78, 81, 88]. Ensuring adequate energy intake, particularly from carbohydrates and fats, is essential to prevent catabolism and promote growth [78, 81]. The Saudi experts recommend regular follow-ups every 6 to 12 months for individuals over one year of age. The GMDI recommends clinical follow-up every one to six months between the ages of 1 and 8 years with monthly biochemical monitoring [36]. Assessment of vitamin B12, zinc, selenium, pre-albumin, iron, and a complete blood count is important to monitor nutritional status and prevent deficiencies [29, 81, 85, 88].

### Emergency and sick day management

Caregivers should be familiar with symptoms of acute metabolic decompensation (AMD) in MSUD, which include poor feeding, malaise, vomiting, dehydration, neurological signs, ketonuria, and ketoaciduria [89, 90]. Emergency or sick day management should start at home and continue in hospital if admission becomes necessary. Early initiation of emergency treatment, including dietary adjustments and intravenous fluids, is essential to prevent metabolic crises [89, 91, 92].

Here, the Saudi experts recommend an emergency or sick day regimen of 0% natural protein for the first 3 days along with isoleucine and valine supplementation, followed by 50% for 2 days, and 100% for 1 day. In general, emergency or sick day regimens for MSUD typically involve a protein-restricted diet along with supplementation of valine and isoleucine [89, 91, 93]. The GMDI guidelines recommend a 50–100% intact protein reduction for 24–48 h and the use of a patient-specific approach [36]. Following the implementation of emergency or sick day management, re-assessment of amino acid levels is crucial to confirm metabolic stability and to adjust treatment as necessary [89, 91, 92].

### VLCAD

#### Nutritional management in below 6 months of age

If VLCAD is suspected, prompt initiation of treatment is advised to prevent metabolic crisis and manage symptoms effectively [30, 94]. Saudi experts recommend that treatment for VLCAD should involve cessation of breastfeeding with the introduction of an medium chain triglycerides (MCT) formula, an approach that facilitates proper fat metabolism and helps patients meet their energy needs [30, 94, 95]. However, breastfeeding in mild asymptomatic VLCAD patients is safe providing they are growing adequately and avoiding fasting between feeds [30, 96]. The use of an MCT formula and avoidance of prolonged intervals between feeds are recommended by the GMDI to help manage energy levels and prevent hypoglycemia [30].

For individuals aged 0–6 months GMDI-recommended fat and energy requirements are shown in Table 11. These are based on adequate levels from the Institute of Medicine’s Dietary Reference Intakes [44]. In infants with VLCAD, emphasis is placed on the avoidance of long-chain fats to meet energy needs [30, 94, 95]. For infants under six months, monthly follow-ups are recommended to monitor nutritional status and overall health [30, 94]. Comprehensive monitoring at each follow-up should include nutritional assessments and comprehensive lab tests, including vitamin and mineral levels, to ensure balanced nutrition and identify any deficiencies early [38].

### Weaning practice

GMDI guidelines suggest that weaning in individuals VLCAD should involve limited intake of dietary fats, continued use of MCT formulas, and follow-ups every 3 months, including nutritional assessments and comprehensive laboratory testing [30]. Restriction of dietary fats, particularly long-chain fatty acids, can help to reduce the build-up of toxic metabolites. Many studies have reported an intake of long-chain fatty acids of 10% of total energy in VLCAD patients; however, severity of VLCAD must be taken accounted for in the extend of restriction [30]. For individuals aged 7–12 months recommended fat and energy requirements are shown in Table 11, as per GMDI.

### Nutritional management in above one year of age

For individuals aged 1–3 years recommended fat and energy requirements are shown in Table 11, as recommended by the GMDI. As with infants, a low-fat diet restricted in long-chain fatty acids and modified to include MCT is recommended to manage energy needs and effectively manage VLCAD [30, 95, 97]. Long-term MCT supplementation should be carefully monitored, since studies in animal models indicate potential adverse effects, such as hepatic steatosis and metabolic syndrome [95, 97]. For individuals aged 1 year and older, triheptanoate, if available, can be used. A retrospective chart review of triheptanoin in long-chain fatty acid oxidation disorders showed that the treatment led to a reduction in hospitalisation and hypoglycaemia events [98]. According to the GMDI, regular follow-ups are recommended with biochemicals monitored every 3–6 months. These follow-ups should involve a comprehensive nutritional evaluation and biochemical monitoring of vitamins, minerals, and overall metabolic status [30]. Omega-3 supplementation is mandatory for these patients.

### Emergency and sick day management

For mild illness, GMDI guidelines state that home management should include frequent high-carbohydrate meals with shorter fasting intervals [30]. Supplementation with medium-chain triglycerides (MCT) is essential to provide an alternative energy source and prevent catabolism [99]. In cases where individuals with VLCAD deficiency are hospitalized and unable to meet their energy needs, it is recommended to administer 10% intravenous (IV) dextrose with electrolytes at 1.5 times the standard maintenance fluid rate [30]. This approach helps to sustain blood glucose levels and prevent metabolic decompensation [99].

### HCU

#### Nutritional management in below 6 months of age

Early detection through newborn screening and the prompt treatment initiation are critical in preventing complications associated with HCU. Treatment should ideally commence within the first few weeks of life, as soon as HCU is suspected [100–102]. Early diagnosis and timely treatment are essential to reduce intellectual disability, skeletal abnormalities, and vascular issues. For infants diagnosed with HCU, a low-methionine diet supplemented with cystine and essential vitamins such as pyridoxine, vitamin B12, and folate is recommended. This typically involves the use of a methionine-free formula to manage plasma methionine and homocysteine levels effectively [100, 101, 103, 104].

Weekly follow-ups are crucial for infants with HCU, allowing for regular monitoring of plasma methionine and homocysteine levels. Formula volume should be adjusted as needed to maintain these target plasma levels [100, 104, 105]. Maintaining plasma homocysteine levels at or below 10 µmol/L is the primary goal to prevent complications [100, 104, 105]. Target plasma total homocysteine levels in the range of 60–100 µmol/L are recommended by the BIMDG; volume of methionine-free formula may be adjusted to meet recommended levels [106].

### Weaning practice

Weaning can be initiated from 6 months of age, as with children without HCU [107]. According to the National Centre for Inherited Metabolic Disorders, first foods for HCU infants should be protein-free fruits and vegetables [107]. Subsequently, weaning should include low protein foods, low protein prescribable products, and methionine-free formula. The European network and registry for homocystinurias and methylation defects (E-HOD) guidelines recommend clinical monitoring frequency to be dependent on age, complications and severity of condition [108]. The BIMDG recommends follow-up every 2 weeks once target levels of total plasma homocysteine and methionine have been achieved [106]. Here, the Saudi experts recommend follow-up every 2–3 weeks, including a review of feeding, in infants aged 6–12 months.

### Nutritional management in above one year of age

A low-methionine diet, supplemented with L-cystine and methyl donors like choline, has been shown to reduce plasma homocysteine levels and help prevent complications, including thrombosis [103, 105]. Methionine restriction is a commonly recommended practice, often accompanied by betaine and vitamins B6, B12, and folate to support homocysteine management [8, 109, 110]. In a cross-sectional survey of 29 metabolic diseases centres in Europe, dietary management was found to vary across centres, however, methionine-free amino acid supplement was prescribed in many patients and many centres recommend cystine supplementation for low plasma levels [111]. Natural protein intake is carefully monitored and adjusted according to age and plasma levels of total homocysteine and methionine [108].

Regular follow-ups every 3–4 months are essential for reviewing dietary adherence and adjusting nutritional intake as needed [104, 111]. Biochemical monitoring, including homocysteine and methionine levels and BCAA, is critical to assess metabolic control and guide dietary modifications [8, 104]. Due to potential deficiencies and the risk of reduced bone mineral density, patients with HCU often require additional calcium and vitamin D3 supplementation [104].

### Role of additions to infant formula for nutritional benefit

Patients with IEM have been shown to be at greater risk of micronutrient deficiency due to the restrictive nature of their diets and therefore supplementation is often necessary [112]. Some amino acids disorders such as PKU, PA, MMA, and MSUD, which are treated with medical formulas containing trace elements, minerals, and vitamins, can achieve adequate micronutrient levels if their intake of the formula is satisfactory [112, 113]. However, patients who consume less medical formula, due to milder disease or even non-adherence, are at greater risk of micronutrient deficiency and therefore may require additional supplementation [113].

Omega-3 can be used for all metabolic patients. A recent systematic review of 11 studies showed that children with IEM often have essential fatty acid deficiency and it is suggested that long-term supplementation with omega-3 polyunsaturated fatty acids are likely to have beneficial effects such as the prevention of cognitive impairment [114]. A study of long-chain omega-3 polyunsaturated fatty acid supplementation in children with PKU showed an improvement in motor skills, measured using the motometric Rostock-Oseretzky Scale, following 3 months of supplementation [115].

On the other hand, GOS, FOS, DHA, and ARA are not generally used unless the patient is severely unwell. To our knowledge there are no studies reporting the use of prebiotics GOS and FOS in patients with IEM [116]. Although low levels of DHA and ARA have been reported in IEM patients with protein-restricted diets, the effect of supplementation on functional outcomes is not known [117]. In infants with VLCAD who require restriction of long-chain fatty acids and MCT supplementation, ARA and DHA supplementation are recommended by the GMDI to prevent deficiency of essential fatty acids [30].

### Challenges in dietary management of metabolic disorders

Poor adherence to diet in IEM patients can result in neurological complications as well as frequent hospitalisations. Patients taking on the responsibility for their own condition after the age of approximately 10 years as well as psychological impact of IEM are thought to contribute to reduced dietary adherence [118]. Educational and cultural background of the patients are also factors affecting adherence to diet [119]. In PKU, 33% of patients struggle to keep their blood phenylalanine levels within the recommended range and this lack of control worsens with patient age [118]. Additionally, feelings of being different in IEM patients of school age can also contribute to poor adherence; family and friends are therefore essential in aiding these individuals to remain adherent to their diet [120].

Attaining nutritional adequacy and meeting growth requirements are key challenges in the management of metabolic disorders. Retrospective analysis of patients with IEM and protein-restricted diets has shown that growth retardation is common in these patients, affecting final height, and is worse during puberty [121]. Additionally, essential amino acid deficiency and subsequent development of malnutrition can also be a challenge for IEM individuals on protein-restricted diets and therefore needs appropriate management [122].

Individuals with metabolic disorders and their caregivers experience significant psychological burden and social isolation. In parent interviews, anxiety, depression, and compassion fatigue were commonly reported, resulting from uncertainty regarding their child’s illness, social isolation, and the demands of looking after their child [123]. Whilst the burden of IEM illness on caregivers is high when their child is young, a shift towards greater burden in children is seen as they get older [124]. Greater psychosocial burden was also observed for children needing to adhere to stricter diets and those in danger of metabolic decompensation [124].

In some centres in Saudi Arabia, a lack of authority of clinical dietitians to order appropriate testing relevant to metabolic diseases is a significant challenge. Whilst clinical dietitians have a significant role in the management of IEM patients, clinicians, such as metabolic geneticists, are responsible for medical management and testing in patients [125].

### Caregiver counselling

Following a diagnosis, the Saudi experts recommend that caregivers should be counselled using a counselling checklist. Initial counselling at diagnosis should focus on providing details of the nature of the disease and it’s manifestations, psychosocial support, immediate management approaches (symptoms of metabolic decompensation, the importance of diet and adherence to diet, preparation of medical nutritional formula), the patient’s individual treatment plan including emergency and sick day regimens, and contact details of the team [125]. Counselling at first follow-up should then focus on discussing the inheritability of the condition, offering carrier testing, encouraging parents to make their relatives aware of the condition due to the familial risk, discuss coping strategies and connecting with other patients as well as support and advocacy groups [125]. Once the patient is older and beginning to manage their own condition, additional counselling is subsequently recommended [125]. Structured counselling sessions are important for building relationships with patients and setting achievable goals [126].

Continuous communication between caregivers and the metabolic centre is imperative, including a call or messaging helpline. Analysis of children with PKU and their families found that bi-directional communication between caregivers or patients and the medical team might help to improve patient outcomes due to greater patient involvement and medical decisions made in the best interest of the patient [119].

Whilst this expert consensus has provided important recommendations for the nutritional management of metabolic diseases in neonates and infants in Saudi Arabia, limitations include the use of a literature search rather than a systematic literature review, which limits the assessment of the strength of each recommendation. Nevertheless, the methodology used here has been used in other expert consensus publications and level of agreement from the experts has been obtained.

## Conclusions

This consensus has provided recommendations for nutritional management of metabolic diseases in neonates and infants in Saudi Arabia using insights and opinions of expert metabolic dietitians from five hospitals across the country. The consensus focused on seven key disorders: PA, MMA, GA1, PKU, MSUD, VLCAD, and HCU. Recommendations have been provided for the nutritional management below six months of age, above one year of age, weaning, emergency nutritional management, and sick day management. These recommendations will facilitate more consistent management of metabolic patients across the country and strive to highlight on-going challenges faced by dietitians, patients, and caregivers. Future work should focus on outcomes associated with dietary management strategies in Saudi Arabia.

## Supplementary Information

Below is the link to the electronic supplementary material.

Supplementary Material 1

## Data Availability

All data generated or analysed during this study are included in this published article.

## Abbreviations

BCAA: Branched-chain amino acids
BCKAD: Branched-chain α-ketoacid dehydrogenase
BIMDG: British Inherited Metabolic Diseases Group
E-HOD: European Network and Registry for Homocystinurias and Methylation Defects
FOS: Fructooligosaccharides
GA1: Glutaric aciduria type 1
GMDI: Genetic Metabolic Dietitians International
GOS: Galactooligosaccharides
HCU: Homocystinuria
IEM: Inborn errors of metabolism
ILE: Isoleucine
LEU: Leucine
MMA: Methylmalonic acidaemia
MSUD: Maple syrup urine disorder
PA: Propionic acidaemia
PHE: Phenylalanine
PKU: Phenylketonuria
SERC: Southeast Regional Newborn Screening and Genetics Collaborative
TYR: Tyrosine
VAL: Valine
VLCAD(D): Very-long chain acyl-CoA dehydrogenase deficiency
