# Supplementary material for: Nutritional management of metabolic disorders in neonates and infants in Saudi Arabia: consensus recommendations
Source: Orphanet J Rare Dis. 2025 Nov 17;20:585. doi: 10.1186/s13023-025-03949-0 (PMC12625242; doi:10.1186/s13023-025-03949-0)
Supplement: Supplementary file 1 — Supplementary Material 1 [file 13023_2025_3949_MOESM1_ESM.docx]

Nutritional Management of Metabolic Disorders in Neonates and Infants in Saudi Arabia: Consensus Recommendations

**Supplementary File**

**Appendix 1.** Biochemical Monitoring of Inborn Errors of Metabolism

**Table A1.1.** Routine biochemical tests performed for each metabolic disorder

| **PA/MMA** | **GA1** | **PKU** | **MSUD** | **VLCAD** | **HCU** |
| --- | --- | --- | --- | --- | --- |
| Amino acids  Pre-albumin  Carnitine  Ketones  Transthyretin  Albumin  CBC  Vitamin D 25-OH | Amino acids  Pre-albumin  Carnitine  Creatinine  CBC | Phenylalanine  Tyrosine  Amino acids  Pre-albumin  Transthyretin  Albumin  CBC  Ferritin  Vitamin D 25-OH | Leucine  Valine  Isoleucine  Amino acids  Pre-albumin  Transthyretin  Albumin  CBC  Ferritin | Creatinine kinase  Carnitine | Amino acids  Pre-albumin  Methionine  Homocysteine (total and free)  Cysteine |

Adapted from GMDI, BIMDG and E-HOD guidelines.

**Appendix 2.** Reference Ranges for Biochemical Monitoring of Inborn Errors of Metabolism

**Table A2.1.** Reference ranges for plasma amino acids

| **Plasma Amino Acids** | **Reference Range in Children (µmol/L)** |
| --- | --- |
| Alanine | 200–450 |
| Alpha-amino-N-butyric acid | 8–37 |
| Arginine | 44–120 |
| Asparagine | 15–40 |
| Aspartic acid | 0–26 |
| Beta-alanine | 0–49 |
| Citrulline | 16–32 |
| Cystine | 19–47 |
| Glutamic acid | 32–140 |
| Glutamine | 420–730 |
| Glycine | 110–240 |
| Histidine | 68–120 |
| Hydroxyproline | 0–5 |
| Isoleucine | 37–140 |
| Leucine | 70–170 |
| Lysine | 120–290 |
| Methionine | 13–30 |
| 3-methylhistidine | 0–52 |
| Ornithine | 44–90 |
| Phenylalanine | 26–86 |
| Phosphoserine | 0–12 |
| Phosphoethanolamine | 0–12 |
| Proline | 130–290 |
| Serine | 93–150 |
| Taurine | 11–120 |
| Threonine | 67–150 |
| Tyrosine | 26–110 |
| Valine | 160–350 |

**Table A2.2.** Reference ranges for CBC

| **Complete Blood Count Reference Ranges in Children (µmol/L)** | | | | | | | | |
| --- | --- | --- | --- | --- | --- | --- | --- | --- |
|  | WBC  (x10^3^ /µL) | RBC  (x10^6^ /µL) | Hematocrit (%) | Hemoglobin (g/dL) | MCV (fl) | MCH (pg) | MCHC (g/dL) | Platelets (x10^3^ /µL) |
| 1m | 6.0–17.5 | 3.0–5.4 | 31–55 | 10.0–18.0 | 85–123 | 28–40 | 29–37 | 150–450 |
| 2m |  | 2.7–4.9 | 28–42 | 9.0–14.0 | 77–115 | 26–34 | 29–37 |  |
| 3-6m |  | 3.1–4.5 | 29–41 | 9.5–13.5 | 74–108 | 25–35 | 30–36 |  |
| >6m – 2y | 6.0–17.0 | 3.7–5.3 | 33–49 | 10.5–13.5 | 70–86 | 23–31 | 30–36 |  |
| 2-6y | 5.5–15.5 | 3.9–5.3 | 34–40 | 11.5–15.5 | 75–87 | 24–30 | 32–36 |  |

**Table A2.3.** Reference ranges for other biochemicals

| **Other Biochemicals** | **Reference Ranges in Children** |
| --- | --- |
| Transthyretin | 18–45 mg/dL |
| Albumin | 3.4–5.4 g/dL |
| Ferritin | Newborns: 25–200 ng/mL  1 month: 200–600 ng/mL  2–5 months: 0–200 ng/mL  6 months and older: 7–140 ng/mL |
| Vitamin D 25-OH | 20–40 ng/mL |
| Free carnitine | <32 days: 15–55 µmol/L  32 days to 12 months: 29–61 µmol/L  13 months to 6 years: 25–55 µmol/L |
| Total carnitine | <32 days: 21–83 µmol/L  32 days to 12 months: 38–73 µmol/L  13 months to 6 years: 35–90 µmol/L |
| Ketones | <0.6mmol/L |
| Creatinine kinase | Male: 39–308 U/L  Female: 26–192 U/L |
| Free carnitine | 23 – 53 µmol/L |
| Total homocysteine | 60–100 µmol/L |
| Free homocysteine | ≤ 10 µmol/L |
| Pre-albumin | 200–400 mg/L |
